# Potential Role of Venular Amyloid in Alzheimer’s Disease Pathogenesis

**DOI:** 10.3390/ijms21061985

**Published:** 2020-03-14

**Authors:** Christopher D. Morrone, Jossana Bishay, JoAnne McLaurin

**Affiliations:** 1 Sunnybrook Research Institute, Biological Sciences, 2075 Bayview Ave, Toronto, ON M4N 3M5, Canada; jossana.bishay@mail.utoronto.ca (J.B.); jmclaurin@sri.utoronto.ca (J.M.); 2 Faculty of Medicine, Department of Laboratory Medicine and Pathobiology, University of Toronto, 1 King’s College Cir, Toronto, ON M5S 1A8, Canada

**Keywords:** venular amyloid, Aβ, vein/venule, Alzheimer’s disease, cerebral amyloid angiopathy, venous collagenosis, perivascular clearance, TgF344-AD rat model

## Abstract

Insurmountable evidence has demonstrated a strong association between Alzheimer’s disease (AD) and cerebral amyloid angiopathy (CAA), along with various other cerebrovascular diseases. One form of CAA, which is the accumulation of amyloid-beta peptides (Aβ) along cerebral vessel walls, impairs perivascular drainage pathways and contributes to cerebrovascular dysfunction in AD. To date, CAA research has been primarily focused on arterial Aβ, while the accumulation of Aβ in veins and venules were to a lesser extent. In this review, we describe preclinical models and clinical studies supporting the presence of venular amyloid and potential downstream pathological mechanisms that affect the cerebrovasculature in AD. Venous collagenosis, impaired cerebrovascular pulsatility, and enlarged perivascular spaces are exacerbated by venular amyloid and increase Aβ deposition, potentially through impaired perivascular clearance. Gaining a comprehensive understanding of the mechanisms involved in venular Aβ deposition and associated pathologies will give insight to how CAA contributes to AD and its association with AD-related cerebrovascular disease. Lastly, we suggest that special consideration should be made to develop Aβ-targeted therapeutics that remove vascular amyloid and address cerebrovascular dysfunction in AD.

## 1. Introduction

### 1.1. Alzheimer’s Disease 

Cognitive decline, memory impairment, and loss of activities of daily living are a few of the debilitating clinical symptoms of Alzheimer’s disease (AD), the most common form of dementia [1,2]. There exists two neuropathological hallmarks characteristic of disease; extracellular deposition of Aβ peptide into amyloid plaques and accumulation of hyperphosphorylated tau into neurofibrillary tangles within neurons [3,4]. Familial AD results from mutations in one of either the *amyloid precursor protein* (APP) or *presenilin-1/-2* genes (PS1/PS2) [5,6]. Overall, mutations in these genes cause an overproduction of Aβ, specifically Aβ40/42, found in parenchymal Aβ plaques [1,4]. Familial AD develops earlier than late onset AD (< 65 years), has a more severe clinical course, and is quite rare, accounting for approximately 1–6% of all AD cases [7]. In contrast, late onset AD is the most common form of AD with onset > 65 years, and a major focus in understanding the possible risk factors that contribute to disease [4,8].

Aβ aggregates in the form of amyloid plaques within the brain parenchyma, typically spreading through the cortex and eventually to the hippocampus [1,9], leading to synaptic loss, neuronal cell death, and brain atrophy [10,11]. This neurodegenerative process contributes to the dementia that is seen in AD patients [12,13,14]. Although Aβ aggregation is one of the major drivers of AD, research has demonstrated cerebrovascular dysfunction to be an important risk factor in late onset AD, occurring earlier than cognitive decline and prior to Aβ deposition [15,16,17,18]. Cerebrovascular morphological alterations, blood–brain barrier (BBB) dysfunction, reduced cerebral blood flow, and decreased vascular reactivity are involved in AD [10,17,18]. Another pathological hallmark of AD that contributes to vascular dysregulation is the presence of Aβ deposition in the walls of cerebral vessels, known as cerebral amyloid angiopathy (CAA) [2]. The consequences of CAA, in combination with cerebrovascular dysfunction, further contributes to dementia [1,10]. Therefore, it is of significant importance to understand the role of the vasculature in AD, as well as elucidate how CAA contributes to disease progression.

### 1.2. Cerebral Amyloid Angiopathy

CAA is one of the most common forms of small vessel disease, defined as the pathological alterations in the small cerebral blood vessels due to accumulation and deposition of a wide variety of amyloid proteins [8,19]. Amyloid proteins are native proteins that undergo a conformational abnormality, causing them to become highly insoluble and prone to aggregation [8,19,20]. In hereditary CAA, there are numerous genetic mutations that give rise to the deposition of several amyloid proteins, leading to severe clinical symptoms [19,20]. The APP Dutch mutation, of the single amino acid substitution at residue 22 of the Aβ protein, is a genetic hallmark of the autosomal dominant condition, hereditary cerebral hemorrhage with amyloidosis (HCHWA)-Dutch type, resulting in severe CAA composing of both wild-type Aβ and the Dutch-type variant [19,20]. HCHWA-Dutch is a fatal disease characteristic of cerebral hemorrhages, stroke, and vascular dementia [19,20,21]. Other genetic variants of the *APP* gene include Swedish (KM670/671NL), Flemish (A692G), Iowa (D694N), and French (V715M) mutations, and also result in a similar clinical presentation as HCHWA-Dutch [8,20,22]. Additionally, mutations in the *PS1* and *PS2* genes contribute to severe CAA [19]. Other genetic modifications include mutations in the *cystatin C*, *transthyretin*, and *gelsolin* genes, resulting in vascular amyloid deposition leading to HCHWA-Icelandic Type, familial amyloid polyneuropathy/meningo-vascular amyloidosis, and familial amyloidosis Finnish type, respectively [19,20]. Hereditary causes of CAA are reviewed in more detail elsewhere [20]. Overall, hereditary CAA is rare, more severe in pathology, and commonly has an earlier age of onset, in comparison to sporadic CAA [8,19,23,24].

In 1938, Scholz was the first to describe CAA in the brains of the elderly [25] and it was not until 1954 that CAA was shown to be distinct from AD, as it was not associated with parenchymal plaques [26]. However, it is important to note the high comorbidities between these disorders, with prevalence of CAA in 80–90% of patients with AD [2,8,19,27]. Moreover, just as the *apolipoprotein E* (ApoE) gene contributes to the disease progression of AD, ApoE is also a genetic risk factor for sporadic CAA and cerebrovascular disease [10]. In particular, ApoE ε2 and ε4 alleles are known to be associated with CAA, with extensive vascular Aβ deposition shown in CAA patients with either allele; whereas ε2 has additionally demonstrated an increased risk of cerebral hemorrhages, despite its known protective effects in AD [8,9,22].

CAA involves the aggregation and deposition of both Aβ40 and Aβ42 peptides [8,19,23,24]. Aβ40 is the predominant isoform in vascular amyloid, the inverse of which is observed in parenchymal plaques; however, evidence suggests that Aβ42 is necessary to initiate CAA deposition [22,28]. Interestingly, the Aβ40:42 ratio in capillaries resembles that in parenchymal plaques and not arterial or venular CAA [22,29]. This suggests that soluble Aβ42 acts as an initial seed of vascular Aβ, but with disease progression Aβ42 deposits into parenchymal plaques. Perivascular clearance of primarily smaller soluble Aβ peptides (i.e. Aβ40) facilitates their deposition along arteries and veins. In support of this, Aβ peptides of 36 to 41 amino acids in length preferentially deposit in CAA, whereas Aβ peptides of 42 and 43 amino acids preferentially deposit in plaques (reviewed in [22]).

It has been of recent interest to understand CAA and its involvement in cerebrovascular diseases, as it is one of the major causes of intracerebral hemorrhages [8,21]. CAA manifests itself in several clinical features such as stroke, leukoaraiosos (i.e. white matter hyperintensities), ischemia, and dementia [30,31,32,33]. Vascular Aβ deposition compromises the cerebrovasculature by weakening blood vessels and increasing vascular resistance, predisposing these vessels to microinfarctions that cause ischemia or hemorrhages [32,34]. Subsequently, vessel rupture and microbleeds occur in cases of CAA regardless of whether patients have AD [22,35]. Since vascular amyloid (i.e. CAA) is involved in most AD cases, it is of great import to understand the role of CAA to gain insight on how vascular amyloid affects the neurovasculature, contributing to the debilitating clinical symptoms in AD patients.

## 2. Impaired Perivascular Clearance of Aβ

There is currently little to no solid evidence of overproduction of Aβ in late onset AD, suggesting that the accumulation of Aβ is due to impairment of clearance [4,8,36]. There are several mechanisms involved in the elimination of small solutes, such as Aβ, from the brain. Of these, the perivascular drainage pathway has a pathological role in AD [24,37,38]. The perivascular drainage pathway is the movement of small solutes along the interstitial fluid (ISF) and into the spaces that surround cerebral microvessels, which are called perivascular spaces or Virchow-Robin spaces [31]. Currently, there are two perivascular drainage pathways that have been proposed in the literature: the intramural periarterial drainage pathway and the glymphatic clearance pathway [39,40].

The intramural periarterial drainage pathway, involves the bulk flow of ISF into the basement membranes of capillaries and arteries that surround smooth muscle cells [4,39,41]. In CAA, amyloid usually deposits along smooth muscle cells of the tunica media and basement membranes of the tunica adventitia of artery walls, which is the site of the periarterial drainage [24,39]. This suggests that poor clearance of this drainage pathway contributes to vascular Aβ deposition and CAA [34,41,42]. In addition, ApoE ε4 has also been associated with reduced perivascular drainage of Aβ along the periarterial pathway [43]. The glymphatic clearance pathway involves the periarterial influx of cerebral spinal fluid (CSF) entering the brain, which combines with ISF and moves along the venous perivascular spaces of large cerebral veins [40,42]. Aquaporin 4 (AQP4) water channels located at the endfeet of perivascular astrocytes largely support the bulk flow of CSF-ISF by facilitating influx of CSF through the periarterial spaces, while promoting clearance of ISF through the perivenous pathway [39,40,42]. Soluble Aβ is one of the solutes that drains along the glymphatic clearance pathway [42] and recent reports demonstrate this perivenous drainage to be impaired in AD [38,39]. We propose that no drainage pathway within the brain acts in isolation and thus, dysfunction of either the periarterial or the perivenous pathways would shift the clearance to the alternate pathway and strain existing mechanisms potentially leading to increased deposition in that blood vessel.

Although both arteries and veins are involved in Aβ drainage, there has been controversy regarding the distribution of Aβ within different vessel walls. Pathological analyses demonstrated that CAA leads to amyloid deposition along arteries and capillaries, with minimal involvement of veins in the AD pathology [24,34]. Human pathological data have been questioned in regards to the lack of consistent methods used to distinguish between arterioles and venules. The reliance on the ratio between the vessel wall thickness to lumen diameter [44] may be flawed as this ratio can be altered due to brain tissue processing [44]. Distinguishing between arterioles and venules is of particular import to correctly identify the type of vessels involved in cerebrovascular pathologies. Accordingly, the pathophysiology of the venous system should not be ignored when studying AD. 

## 3. Evidence of Aβ in Veins and Venules

The venous system of the cerebrovasculature has had scarce attention in regards to understanding the mechanisms involved in CAA, and, in association, AD [33]. Recently, there has been increased importance in studying the pathology of veins and venules, not only in the context of AD or CAA, but also in small vessel disease and dementia overall [30,45,46]. Venous pathology has been shown to contribute to vascular dysfunction in AD, resulting in white matter hyperintensities and microinfarcts, and potentially induction of ischemia [30,45,46,47,48]. However, more research is necessary to understand the role of veins and venules in AD and CAA. One controversial pathology of the venous system is the presence of venular amyloid, the deposition of Aβ within veins and venules. As such, we discuss evidence for the presence of amyloid in veins and venules, suggesting that all vessels play a role in disease progression [27,49,50,51]. In addition, numerous reports demonstrate a suite of mechanisms that involve a pathological role for veins in CAA and AD [27,30,47,50,51,52]. This section outlines the evidence for venular amyloid in both animal models and clinical cases, in the context of AD and CAA.

### 3.1. Preclinical Models

Currently, in vivo animal studies have observed venular amyloid. The APP+PS1 rat model, that shows extensive CAA, Aβ plaque formation, and behavioural alterations, demonstrated Aβ deposits in leptomeningeal and cortical arteries, cortical capillaries, as well as severe deposition in the leptomeningeal and cortical veins using immunohistochemical analyses [53]. In addition, there were significant alterations in the veins including enlarged cortical veins and perivascular spaces, and stenosis of collagen, tau, and Aβ [53]. These pathological features have also been identified clinically in venous insufficiency and vasogenic edema, typically depicted as hyperintensities in magnetic resonance imaging (MRI) [30,31,47,52,54,55].

**Figure 1:**
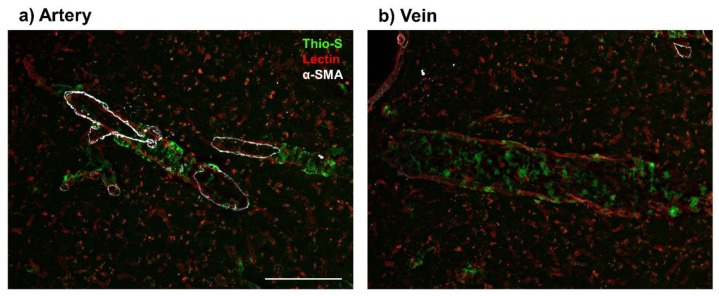
Presence of venular amyloid in 16-month old TgF344-AD rats. Immunofluorescence imaging of vascular amyloid deposition, stained by Thioflavin S (Thio-S, green), in cortical penetrating vessels (Lectin, red). Amyloid beta peptide (Aβ) deposits present a ‘double-barreling’ morphology, forming cyclical rings surrounding the arteries (**a**). Venular Aβ is present as smaller, globular deposits along the veins (**b**). Arteries were distinguished from veins by presence of alpha smooth muscle actin (α-SMA, white). Scale bar = 100 μm.

Further studies identify Aβ in veins of TgF344-AD rats, that overexpress human Swedish *APP* and *PS1* with exon 9 excised [56]. TgF344-AD rats recapitulate AD progression in humans including age-dependent cerebral amyloidosis, CAA, tau pathology, neuronal loss, and cognitive dysfunction [56]. In studying early neurovascular dysfunction, Joo and colleagues demonstrated that vascular Aβ deposition was not exclusive to arterioles and can be found in venules to a lesser degree, at 9 months of age [18]. Moreover, TgF3444-AD rats have a 51% decrease in vascular reactivity in venules in response to hypercapnic conditions [18]. Here we show amyloid deposition in the veins of TgF344-AD rat models at 16 months of age (Figure 1). The topography of these amyloid deposits are quite distinct from deposition in arteries (Figure 1a); where venous Aβ deposits appear more globular and have a patchy distribution (Figure 1b), whereas arteriolar Aβ possessed a ‘double-barreling’ morphology, forming striped rings, which is in correspondence with previous literature [19,27,57]. Since Joo and colleagues showed significantly less amyloid deposition in veins and venules relative to arteries and arterioles [18], we propose an age-dependent association between arterial (Figure 1a) and venular amyloid (Figure 1b). However, further research is necessary to understand the interplay between these amyloid deposits as age and AD progresses.

Mouse models have demonstrated impairment and/or suppression of both the periarterial drainage pathway and the glymphatic clearance pathway in AD [37,38,58]. Intracerebral injections of a dextran tracer were performed in 3-, 7-, and 22-month old Tg2576 mice, a model of AD overexpressing the human Swedish APP mutation [37]. Hawkes and colleagues demonstrated that a 3-kDa dextran almost exclusively drains along the basement membranes of capillaries and arteries in non-transgenic mice, with little to no drainage in veins [37,41]. In agreement, an AD mouse model with the overexpression of the London mutation within the human *APP* gene exhibited significant Aβ deposition along the walls of arteries but sparse deposition along veins [59]. However, most striking in the Tg2576 AD mice, not only were capillaries and arteries positive for dextran in all ages, but dextran-positive staining along veins was detected at 22 months of age [37]. These results suggest that Aβ deposition in arteries and capillaries can exacerbate periarterial drainage altering the nascent clearance route of solutes from the brain [37]. An alternate explanation for the presence of dextran, and potentially Aβ, along veins may be impaired glymphatic drainage with aging [38,58]. 

One report demonstrated venular amyloid in APP/PS1/Cx3cr1 mice presenting as small, globular Aβ aggregates in veins at 5 months of age and at 9 months of age, along with the presence of the ring-like Aβ deposition in arteries [27]. Michaud and colleagues proposed that the Aβ deposits in veins precede arterial Aβ deposition [27], which is contrary to previous findings [18,37,53,59]. In addition, Michaud and colleagues demonstrated preferential adherence of monocytes to surrounding Aβ-laden veins, but not arteries, and that these monocytes internalize venular amyloid for removal into the bloodstream [27]. This work proposes underlying mechanisms that contribute specifically to clearance of Aβ via veins and venules and further raises the question about the knowledge gap regarding the relationship between Aβ deposition in arteries/ arterioles versus veins/ venules.

Preclinical studies showed venular amyloid in multiple models of AD but was observed to a lesser extent compared to arterial CAA. We previously proposed two models that may explain the lower presence of amyloid in veins and venules, despite the important role of veins in the glymphatic drainage system [39]. The first is that the lack of the highly organized smooth muscles cells in veins and venules result in less substrates for Aβ deposition and, secondly, monocytes that exclusively adhere to veins might clear Aβ as it deposits along the venular walls [4,27,39]. In addition to the preclinical models discussed above, there are reports that do not distinguish between arteries and veins [60,61,62,63,64], or exclusively report of CAA in arteries [65,66]. Therefore, the present findings in this section address the need for characterization of all cerebral vessels to accurately gain a comprehensive understanding of both arterial and venous CAA. 

### 3.2. Clinical Evidence

Cases of venular amyloid deposition have been reported as early as the 1980s, where 45 patients were studied to determine the relationship between vascular amyloid and amyloid plaques [49]. The proportion of Aβ deposition in each vessel was recorded according to a severity measurement, 0–4, now termed as the Mountjoy scale [49,50]. Mountjoy and colleagues determined the presence of amyloid not only in arteries, but in veins of 21 patients, as depicted by Congo Red staining, with one case having severe amyloid deposition in veins [49]. 

Weller and colleagues also demonstrated Aβ deposition in veins, although to a lesser extent compared to arterial Aβ deposition [24,34]. In one case study, 17 AD brains were examined to determine Aβ deposition in accordance with perivascular drainage. The leptomeningeal vessels were stripped from the surface of the brain and were stained with Thioflavin S to quantify amyloid deposition in 681 arteries and 352 veins. Aβ deposition demonstrated a 5:1 distribution in arteries compared to veins, with approximately 4.5% of veins positive for amyloid [34]. In a clinical case study of AD, Weller and colleagues also observed Aβ deposition in the walls of veins in the presence of severe CAA [24]. As very little Aβ deposits were detected in these veins, Weller and colleagues considered these deposits as rare, and attributed the CAA pathology to impaired clearance of the periarterial drainage pathway [24]. Current disease models suggest that even when rare, venular Aβ can impair glymphatic clearance, contribute to Aβ deposition, and exacerbate CAA and AD progression [22,24,34,39,40,42].

In contrast, a neuropathological study of 69 human autopsy brains assessed the distribution of Aβ deposition across different vessels and the association with pathological features such as CAA severity and AD-related Aβ pathology [51]. Two types of CAA were identified; CAA Type 1 and CAA Type 2, which were associated with the presence and absence of capillary Aβ deposition, respectively. An important observation in this study was that both CAA types showed Aβ deposition in leptomeningeal and cortical veins, suggesting that CAA is not exclusive to arteries, but also in veins and differentially in capillaries [51].

A recent paper examined the potential venular involvement in intracerebral hemorrhages [50]. All 41 patients showed the presence of CAA, as demonstrated by both Congo red and anti-Aβ(8–17) immunohistochemical staining. The severity of the vascular Aβ deposition was determined by the Vonsattel scale and the proportion of Aβ in each vessel was recorded based on the Mountjoy scale [49]. Of the 41 cases, 33 patients had Aβ deposits in veins, 15 of which showed severe CAA, 13 showed moderate CAA, and the remaining 5 patients showed mild CAA [50]. In light of the involvement of moderate and severe CAA associated with venular amyloid deposition, these data support our hypothesis that dysfunction of either the periarterial or the perivenuous pathways would shift clearance to the alternate pathway and strain existing mechanisms potentially leading to increased deposition in that blood vessel.

Overall, venular amyloid has been understudied with respect to vascular mechanisms involved in AD. Despite this, the involvement of venous and venular pathology in cerebral microinfarction and other cerebrovascular diseases have indicated that veins and venules play a strong role in contributing to dementia [30,46]. Moreover, compromise in the functional role of the venous system could in part be a major factor in impairing solute clearance from the brain, leading to vascular Aβ deposition [16,30]. Therefore, studies elucidating the relationship between pathology in veins and venules with Aβ deposition is of high interest.

## 4. Contribution of Venular Amyloid to Alzheimer’s Disease (AD) Pathogenesis

**Figure 2:**
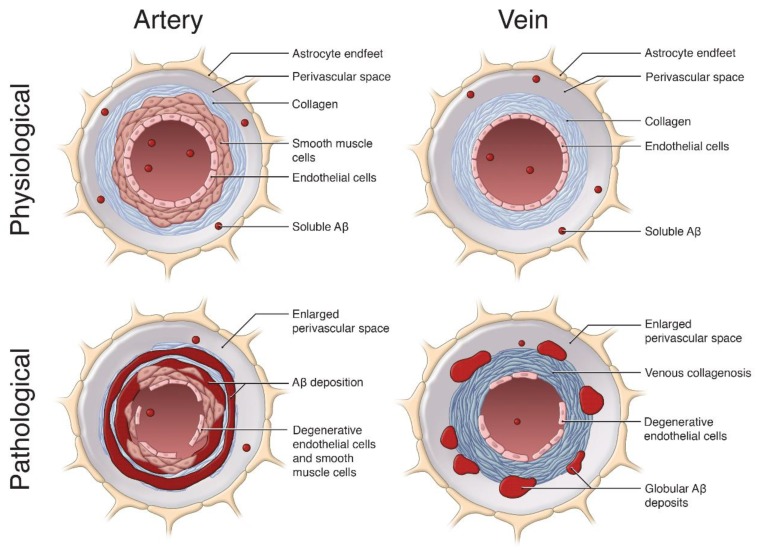
The pathological role of vascular Aβ deposition on arteries and veins. This schematic summarizes the effects of Aβ (red) accumulation on cerebral vessels, relative to physiological conditions. In physiological conditions, soluble Aβ can be cleared from the brain into the vessel lumen and along the perivascular spaces of arteries and veins. In pathological arteries, Aβ is deposited along the smooth muscle cells and basement membranes, forming a ‘double-barreling’ morphology surrounding the vessel, resulting in arterial smooth muscle and endothelial cell loss. In pathological veins, the Aβ deposition is distinct, forming globular deposits in perivascular spaces. Venular Aβ contributes to extensive venous collagenosis (dark blue) in the form of concentric rings along venular walls, significant enlargement of the perivascular space, and endothelial cell loss. These pathological features in arteries and veins exacerbate Aβ accumulation, ultimately causing vascular and cognitive dysfunction in cases of cerebral amyloid angiopathy (CAA) and Alzheimer’s disease (AD). Image not to scale. Image by Sherry Lai.

In this review so far, we have proposed that accumulation of Aβ along the venous system occurs to a greater degree than previously thought. Venular amyloid is likely an integral part of AD pathogenesis, and similar to arterial amyloid, contributes to cerebrovascular dysfunction and failure of Aβ clearance mechanisms (Figure 2). In this section, we will discuss how venous collagenosis, arterial and venular pulsation impairments, and enlargements in the perivascular space may contribute to the accumulation of venular amyloid, and vice versa (Figure 2). We propose that Aβ deposition in the venular system acts in a positive feedback manner, similar to arterial CAA, to increase vascular Aβ deposition from impaired ISF efflux, and impair the cerebrovascular network.

Microinfarcts and small vessel disease commonly occur in AD patients, impairing cognition and increasing risk for stroke [30,67]. Recently, blockage of a single venule in mice vastly impaired cerebrovascular structure and function [46,68]. Acute venular occlusion in the mouse cortex decreases blood flow, increases vessel diameter, impairs local neuronal function and neurovascular coupling, induces severe hypoxia, and can cause somatosensory behavioural deficits [69,70,71]. Interestingly, even though mice have 2-3x more cortical penetrating venules than arterioles, the occlusion of a single venule had a profound impact, indicating the potentially compounding effect of venular amyloid [46,68,72]. Conversely, humans have less venules for every arteriole [46], suggesting a greater strain on each individual venule. Therefore, venular amyloid, with the associated collagenosis and vascular impairments in AD, likely accelerates disease progression.

### 4.1. Venous Collagenosis

Venous collagenosis was first described in the mid 1990s as a non-inflammatory mural disease and collagenous thickening of the walls of periventricular venules and veins, associated with cases of leukoaraiosis and with aging [52,54]. Leukoariosis, also referred to as white matter hyperintensities, are MRI- and computed tomography-detectable markers of cerebrovascular dysfunction [30,54]. Moody and colleagues discovered collagenosis in the venules of 65% of people over 60 years of age histologically by excessive collagen accumulation and the replacement of venular mural cells with collagen. Vein and venule walls were thickened and exhibited concentric rings of collagen [52,54]: specifically, collagen 1 and 3 accumulated excessively in venular walls, indicative of hyalinization [16,73]. Collagenosis can occlude veins in individuals with leukoaraiosis, even in those who present with normal afferent vessels, and have profound effects on the cerebrovascular system, including impairment of perfusion pressure and blood flow [46,52,54]. Moody and colleagues originally observed collagenosis specifically on efferent vessels, not on capillaries or arterioles, which was most severe and abundant in the periventricular venular walls [52,54]. However, venous collagenosis was also detected in connected regions and in the cortex [54,73], suggesting that additional triggers such as amyloid could exacerbate collagenosis throughout the brain.

More recently, Keith and colleagues examined brains of AD patients for periventricular venous collagenosis and venous stenosis both of which positively correlated with and predicted the severity of white matter hyperintensities, a common AD-related cerebrovascular dysfunction [47,74]. Additionally, MRI images of periventricular white matter hyperintensities demonstrate the presence of venules within these lesions [30,55], suggesting an association with venous collagenosis. Keith and colleagues separated veins into small (<50 μm diameter), medium (50–150 μm diameter), and large ( > 200 μm diameter) vessels. Venous walls appeared thickened by the presence of collagen, and small and medium veins exhibited a greater collagen:lumen ratio, which positively correlated with severity of white matter hyperintensities. Large veins with prominent collagenosis were selected and percent stenosis was assessed by comparing the internal vessel diameter with the external (includes collagenous deposits) [47]. Overall, significant collagenosis and stenosis of the venous system, in particular in the deep medullary veins, occurred in AD patients [47]. In combination with the results described by Moody and colleagues, these data indicate that venous collagenosis is enhanced by both age and AD-related pathology, such as white matter hyperintensities, and can impair cerebrovascular structure, cause venous insufficiency, increase fluid leakage/vasogenic edema, and occlude blood flow [30,46,47,52,54,55,75,76,77]. 

Evidence of venous collagenosis in AD preclinical models is limited to a single report in the APP+PS1 rat model, which present with severe cortical and leptomeningeal venular Aβ deposition [53]. Subsequently, these rats recapitulate collagenosis of the venous system as it was described in humans [47,52]. In APP+PS1 rats, venous collagenous leptomeningeal, cortical, hippocampal and cerebellar deposits and concentric stenosis associate with Aβ deposition [53]. Enlargements in the area of vascular and perivascular spaces, as well as increased neuronal necrosis in the surrounding area demonstrate the extent of cerebrovascular impairments [53], which can contribute to functional deficits and to an increased Aβ deposition from impaired clearance mechanisms [30,31,40,78].

The mechanisms by which venular amyloid deposition or venous collagenosis begin are not entirely understood. However, it is possible that glymphatic clearance of CSF-ISF along veins allows soluble Aβ to deposit in perivascular spaces and venular walls, similar to what is proposed for arterial CAA. This in turn will impair cerebrovascular function and exacerbate hyalinization of venules [42,46,73,78]. Interestingly, venous collagenous deposits in APP+PS1 rats were positive for the presence of Aβ and tau in immunohistochemical staining [53], suggesting that the deposition of Aβ may increase the deposition of collagen on venules, and vice versa. Additionally, oxidative stress and leukocyte adhesion have been implicated in the initiation of venous collagenosis, which are reviewed in [46]. Briefly, monocytes, which are known to adhere to Aβ-positive veins, can release reactive oxidative species that impair the venous system [27,46,79]. Finally, as a result of venular amyloid, collagen deposition will initiate structural and functional alterations in these vessels (Figure 2), specifically enlargements in the perivascular space, and alterations in pulsatility and blood flow, culminating in impaired perivenous Aβ clearance which further exacerbates vascular Aβ deposition and drives AD pathogenesis.

### 4.2. Cerebrovascular Pulsatility 

Blood vessel wall rigidity and stiffness decrease blood flow through the cerebrovascular system. AD-related arterial CAA and venous collagenosis increase afferent vessel pulsatility and efferent vessel resistance, respectively [30,34,54]. Arterial Aβ stiffens vessels and forms highly rigid vessel walls, thereby increasing pulsatility [34,80,81]. When this high-pressure blood flows into the AD venous system it is met with high resistance from amyloid and collagen deposits [30,53,54], causing physical stress on venules [82]. Criswell and colleagues investigated arterioles and venules in aged canine brains and determined how regional differences in vessel structure impacts venular resistance and increased venous collagenosis [83]. Specifically, periventricular white matter venules demonstrated approximately half the intramural cells when compared to subcortical white matter venules, indicating a lower baseline vessel wall resistance. Because of lower resistance, there is an increased susceptibility to stress from arterial pulsatility in periventricular venules, offering a potential explanation for severe age-related collagenosis in these vessels [52,83]. In the context of AD-like pathology, it is possible that this effect is further exacerbated by arterial and venular Aβ [34,51], since Aβ40 is directly toxic to pericytes in vitro [84,85,86]. Loss of venular mural cells, which are primarily pericytes, has been well demonstrated in AD rodent models [18,45], and mural cell loss associates with cognitive decline in AD patients [87,88].

Furthermore, cerebrovascular Aβ directly correlates with impairments in vessel pulsation and resistance in AD [81]. Utilizing transcranial color-coded duplex sonography, Ortner and colleagues determined that high pulsatility and resistance indices in the middle cerebral arteries associated with increased cognitive impairments and positive amyloid status in AD and mild cognitively impaired patients, compared with healthy controls. This effect was independent of structural microvascular damage (i.e. atherosclerosis) as no decreases were detected in the retinal arteriolar-venular ratio [81]. However, it is possible that narrowing of arterioles as well as venules, from amyloid and collagenosis [34,54], caused the ratio to remain the same as in healthy controls. In support of this, AD patients exhibit increased pulsatility and resistance in a variety of both afferent vessels, including the middle cerebral artery and internal carotid artery, and efferent vessels, including the superior sagittal, straight and transverse sinuses, visualized with 4D flow MRI [89,90]. 

Recently, van Veluw and colleagues demonstrated that clearance of an injected fluorescein dextran can be enhanced by evoking arterial vasomotion at 0.1 Hz, with visual stimuli. This frequency of vasomotion occurs spontaneously in arterials, and correlates with ISF clearance [91,92,93]. Interestingly, AD mice with CAA do not exhibit enhanced clearance from evoked vasomotion, likely as a result of Aβ-related arterial stiffness, tortuosity and impaired pulsatility and resistance indices [16,57,81,89,90,91]. The authors did not observe 0.1 Hz spontaneous vasomotion in venules [91]; however, venular vasomotion from longer stimulations and/or a delayed response to arterial blood flow [92] may associate with and enhance perivenous ISF clearance [42,78]. Glymphatic perivenous clearance of the MRI contrast agent gadobutrol has recently been documented in humans. Meng and colleagues delivered gadobutrol to the ISF with MRI-guided focused ultrasound and observed clearance through the venular perivascular space, subarachnoid space and around draining veins [94]. Overall, vasomotive forces drive ISF clearance, and are impacted by Aβ accumulation and AD-related cerebrovascular dysfunction. With venular amyloid and collagenosis there are dysfunctional vessel pulsations and decreased blood flow through the brain, which contribute to reduced periarterial and perivenous ISF solute clearance with AD progression.

### 4.3. Enlargements in the Perivascular Space 

Soluble Aβ in the ISF clears from the brain within arterial and venous perivascular spaces (also referred to as Virchow-Robin spaces) which surround small cerebral vessels [31,39,41,42,78]. Flow through these fluid-filled compartments is impeded by perivascular accumulation of cellular debris, as well as from amyloid and collagen aggregates on vessel walls. A loss of perivascular AQP4 channels occurs in AD patients, indicating reduced glymphatic CSF-ISF exchange and flow [31,95,96,97]. Finally, perivascular spaces are enlarged with age and AD pathology, and become visually detectable on MRI scans [31,98,99].

Banerjee and colleagues suggest that MRI-visible perivascular spaces in Alzheimer’s disease are a result of cerebrovascular dysfunction, specifically small vessel disease co-morbidities, as they observed no association between amyloid burden and perivascular space enlargement [99]. However, others have reported strong associations; amyloid positron emission tomography (PET) and histopathology measurements colocalized with enlarged cortical perivascular spaces in cases of CAA and AD [97,98,100,101]. Support for this is seen in APP+PS1 rats, which exhibit a 1.7-fold increase in the perivascular space along frontal cortical vessels. Since this is an Aβ-driven model with venular amyloid, enlargements in the perivascular space are likely driven by Aβ [53]. Additionally, tortuosity, a common cerebrovascular structural abnormality common in leukoaraiosis and AD, creates cavities in the surrounding brain parenchyma [16], potentially contributing to enlarged perivascular spaces. In the TgCRND8 mouse model of AD, arterial tortuosity occurs as a direct result of arterial Aβ deposition [57], and venular tortuosity occurs from pharmacological depletion of mural cells, which are lost in AD patients [45,87,88]. Therefore, vascular amyloid accumulation contributes to the enlargement of perivascular spaces, and through impaired Aβ clearance, enlarged perivascular spaces likely enhance vascular Aβ deposition.

Venular amyloid and associated dysfunction in the venous system, including collagenosis, high pulsatility and resistance indices, and enlarged perivascular spaces, impair the efflux of ISF and Aβ from the brain, and thereby act in a positive-feedback loop exacerbating Aβ deposition and vascular dysfunction (Figure 2). Considering that vascular deficits are common co-morbidities of AD [16,30], there is a need for effective AD therapeutics that target the removal of vascular amyloid.

## 5. Limited Efficacy of Aβ-Targeted Therapeutics on Vascular Amyloid

Aβ-targeted treatments have been a focal point of AD therapeutic design for the last two decades. Despite some recent success with Aducanumab reducing cognitive decline, this class of therapeutics have been limited in disease modifying and clinically beneficial effects. Potential explanations include that Aβ-targeted therapeutics were characterized in primarily Aβ-driven preclinical models, targeted too late in disease progression in AD patients, and that the contributions of tau pathology to neurodegeneration and cognitive impairment persist with Aβ attenuation [13]. However, one mostly overlooked factor is that most Aβ-targeted therapeutics do not remove vascular amyloid.

The first Aβ immunotherapy to enter the clinic for AD was AN-1792, which involved active immunization with the full-length Aβ peptide. AN-1792 led to acute and long-term reduction of parenchymal Aβ plaques, exhibiting almost complete plaque clearance 14 years after treatment [102,103]. However, vascular amyloid was not attenuated and there was persistent and severe CAA in most patients [102,103]. Cerebral cortical and leptomeningeal vessel walls exhibited more complete Aβ coverage and a significantly greater number of Aβ42 positive vessels (14× and 7× more, respectively) in immunized compared to non-immunized AD patients [104]. Interestingly, Aβ42 is normally absent in vascular amyloid deposits, and therefore these results suggest that Aβ is solubilized from parenchymal plaques and accumulates along blood vessels during ISF clearance [41,42,104,105]. Conversely, Bapineuzumab passive immunization of an antibody to the same Aβ antigenic site in AD patients reduced CAA on amyloid PET imaging, but was associated with a large degree of amyloid-related imaging abnormalities and vasogenic edema, with no clinical cognitive benefit [106,107,108], reviewed in [109]. Considering how venular amyloid-related impairments (i.e. collagenosis, impaired blood pulsation/flow, enlarged perivascular spaces) can impair clearance and increase vascular Aβ deposition, Aβ-targeted therapeutics going forward should be designed to reduce vascular amyloid as well. 

The only passive Aβ immunotherapy directly investigated for CAA was Ponezumab, which specifically targets vascular Aβ. Ponezumab is a monoclonal antibody against Aβ40 [110,111], which is the primary Aβ species in CAA [19,105]. Initial results in AD rodent models with CAA were promising, including the reduction of Aβ-positive leptomeningeal and cortical vessels, reduced vascular soluble Aβ40 and Aβ42, improved hypercapnic response, and only transiently increased Aβ40 in the ISF, indicating functioning perivascular/glymphatic Aβ clearance [110]. However, in cases of probable CAA, Ponezumab treatment exacerbated impairments in cerebrovascular reactivity in response to visual stimuli, measured by blood–oxygen-level-dependent functional MRI signal in the visual cortex [111]. CAA was not assessed, but results suggest that target engagement was limited [111,112]. The Ponezumab results reveal the complicated nature of targeting vascular Aβ in CAA and AD, and demonstrate the potential need for alternatives to Aβ immunotherapy.

Another class of Aβ-targeted therapeutics are beta-site APP cleaving enzyme 1 (BACE1; β-secretase) inhibitors which reduce the production of Aβ [113]. *BACE1* is expressed in cerebrovascular associated cells on leptomeningeal and cortical vessels, including smooth muscle and endothelial cells [114]. Furthermore, leptomeningeal vessels extracted from patients with CAA exhibit elevated BACE1 levels and activity [114]. Thus, data demonstrate a potential role of BACE1 in vessel-localized Aβ production and vascular Aβ deposition. The BACE1 inhibitor NB-360 attenuates Aβ in preclinical AD models [115,116], and was recently investigated in the APPDutch CAA mouse model [117]. APPDutch mice develop leptomeningeal and cortical CAA composed primarily of Aβ40, without exhibiting parenchymal plaques. It is unconfirmed whether venular amyloid is present in this model [117,118]. NB-360 treatment at the age of CAA onset reduced score, frequency, and severity of CAA measured by immunohistochemistry, with a 90% reduction of CSF Aβ [117]. Importantly, once cleared from the blood vessels, Aβ was not deposited in parenchymal plaques [117]. Smooth muscle cell loss was attenuated and microhemorrhages were not exacerbated by NB-360 treatment [117,119], indicating rescue of amyloid-associated cerebrovascular impairment. More work is needed to determine the efficacy of NB-360 as a therapeutic intervention when CAA has already accumulated. Currently, BACE1 inhibitors have failed to improve cognition in AD patients [120], and therefore these results demonstrate the potential role for BACE1 inhibitors specifically in cases of CAA and warrant further investigation. 

## 6. Conclusions

Most cases of AD exhibit presence of cerebrovascular amyloid (i.e. CAA) and are often co-morbid with cerebrovascular diseases [8,30]. To date, research has attributed CAA to pathology in arteries and arterioles, with veins having a minimal role [24,34]. However, several papers have presented data demonstrating vascular amyloid in veins and venules in both preclinical animal models and clinical case studies [27,50,51,53]. In preclinical models, the presence of venular amyloid, in combination with the decreased vascular reactivity [18] and venular degeneration [45] further supports the potential role of the draining vessels in AD. 

Despite the differences in preclinical experiments, clinical studies of venular amyloid provides evidence for the involvement of veins in CAA severity and AD [24,34,50,51]. We highlight several mechanisms that may increase venular amyloid deposition and contribute to venular pathology. Venous collagenosis, impaired cerebrovascular pulsatility and vasomotion, and enlarged perivascular spaces contribute to AD-related cerebrovascular dysfunction and may be associated with arterial and venular amyloid, suggesting further disturbance in the clearance of Aβ along the perivascular drainage pathway (Figure 2) [30,47,53,54,81,99]. Overall, we propose that these mechanisms act in a positive-feedback loop, promoting venular Aβ deposition and vice versa.

Herein, we propose venular amyloid is an integral part of AD pathology, and that numerous mechanisms related to the pathophysiology of veins (i.e. venous collagenosis, impaired cerebrovascular pulsatility, enlarged perivascular spaces) may lend further insight into the cerebrovasculature dysfunction in AD. This critical role of vascular pathology could help explain the limited efficacy of Aβ-targeted therapeutics, as they typically do not remove vascular amyloid. Improvements in arterial and venular classifications, appropriate choices of preclinical models and treatment design oriented towards removing vascular Aβ, will benefit CAA and AD research going forward. Recognizing the importance of venular, as well as arterial, amyloid in AD pathogenesis will be integral to increase treatment specificity and efficacy, and to gain a comprehensive understanding of disease progression.

## Abbreviations

α-SMA	alpha-smooth muscle actin
Aβ	amyloid-beta
AD	Alzheimer’s disease
APP	amyloid precursor protein
AQP4	aquaporin 4
BACE1	beta-site amyloid precursor protein cleaving enzyme 1
BBB	blood–brain barrier
CAA	cerebral amyloid angiopathy
CSF	cerebrospinal fluid
HCHWA	hereditary cerebral hemorrhage with amyloidosis
ISF	interstitial fluid
MRI	magnetic resonance imaging
PET	positron emission tomography
PS1/PS2	presenilin-1/ -2
